# Harmless Treatment of High Arsenic Tin Tailings and Environmental Durability Assessment

**DOI:** 10.3390/ijerph191811247

**Published:** 2022-09-07

**Authors:** Weiwei Zhao, Zhengfu Zhang, Hui Yang, Xian Zhou, Jinsong Wang, Chengping Li

**Affiliations:** 1 Faculty of Materials Science and Engineering, Kunming University of Science and Technology, Kunming 650093, China; 2 Kunming Metallurgical Research Institute Co., Ltd., Kunming 650031, China

**Keywords:** arsenic, environmental durability, mechanism of stabilization/solidification, tin tailings (TT)

## Abstract

The treatment of arsenic (As) in tin tailings (TT) has been an urgent environmental problem, and stabilization/solidification (S/S) treatment is considered an effective technology to eliminate contamination of As. In this study, we developed a low-carbon and low-alkalinity material to S/S of As, and the results showed that the leaching concentration of As after treatment was lower than the Chinese soil environmental quality standard (0.1 mg/L). Based on a series of characterization tests, we found that OH^−^ promoted the dissolution of As(III)-S, Fe-As(V), and amorphous As(III)-O species and formed Ca-As(III) and Ca-(V) species with Ca^2+^. Simultaneously, hydration produces calcium silicate hydrate (C-S-H) gel and ettringite by the form of adsorption and ion exchange to achieve S/S of As. We also assessed the durability of this material to acidity and temperature, and showed that the leaching concentration of As was below 0.1 mg/L at pH = 1–5 and temperature 20–60 °C. The method proposed in this study, S/S of As, has excellent effect and environmental durability, providing a new solution for harmless treatment of TT and its practical application.

## 1. Introduction

The rapid development of industry has greatly improved people’s material life; simultaneously, the awareness of environmental protection has gradually increased. Especially in the development and utilization of mineral resources, a huge amount of low-utilization tailings are often produced, which not only take massive valuable land resources but also the harmful elements such as arsenic (As) in the tailings will spread to the surrounding soil and water sources over time, which will eventually endanger human life and health through the action of the food chain [1,2].

As is a metallic element that is widely distributed in the Earth’s crust and can be As^3−^ (arsine), As^0^ (arsenic monomers), As^3+^ (arsenite), and As^5+^ (arsenate) valence states. As monomers exist in three isotopes: gray As, yellow As, and black As [3]. Gray As is the most common As monomer, a bilayer structure, which is composed of many interlocked vertical six-membered rings with weak bonding between the layers [4,5]. The As^3+^ form and the As^5+^ form are the most common valence states, and inorganic compounds consisting of As^3+^ are more toxic than As^5+^ [6]. Currently, the use of As is limited and is commonly used as wood preservation, pesticides, etc. [7]. As in minerals is mainly in the form of sulfides such as arsenopyrite (FeAsS) [8], realgar (As_4_S_4_) [9], and orpiment (As_2_S_3_) [10], and these compounds are often found in tailings and are relatively stable in strong alkaline and reducing environments [11]. Under the action of natural weathering conditions, As will be released into the environment [12]. The process of FeAsS oxidation reaction is shown as follows:

(1) $${4\mathrm{FeAsS}+11O}_{2}{+6H}_{2}O\to {4\mathrm{Fe}}^{2+}{+4H}_{3}{\mathrm{AsO}}_{3}{+4\mathrm{SO}}_{4}{}^{2-}$$

(2) $${2H}_{3}{\mathrm{AsO}}_{3}{+O}_{2}\to {2H}_{2}{\mathrm{AsO}}_{4}{}^{-}{+2H}^{+}$$

(3) $${4\mathrm{Fe}}^{2+}{+O}_{2}{+4H}^{+}\to {4\mathrm{Fe}}^{3+}{+2H}_{2}O\;$$

As_4_S_4_ is easily converted to As_2_O_3_ under oxidation conditions. The process of As_2_S_3_ oxidation reaction is shown as follows:

(4) $${\mathrm{As}}_{4}S_{4}{+7O}_{2}\to {2\mathrm{As}}_{2}O_{3}{+4\mathrm{SO}}_{2}\;$$

(5) $${2\mathrm{As}}_{2}S_{3}{+3O}_{2}\to {2\mathrm{As}}_{2}O_{3}+6S↓$$

(6) $${\mathrm{As}}_{2}O_{3}{+3H}_{2}O\to {2H}_{3}{\mathrm{AsO}}_{3}$$

Under different weathering conditions, the oxidation products of As are different, but most of them are produced in the form of arsenate. Oxidation of As-S minerals such as FeAsS will result in an increase in the leaching of As from the tailings, which poses a serious hazard to the surrounding environment [13]. Studies have shown that the reaction of iron and calcium salts with arsenate ions produces the corresponding precipitates, which can effectively reduce the leaching of As from tailings [14,15,16]. However, there are some differences in the composition and types of As-containing minerals in different types of tailings or the same type of tailings from different regions [9]. Current studies have generally focused on the study of As in gold tailings, but little attention has been paid to the species and transformation processes of As in tin tailings (TT), and different species of As may have different effects on the immobilization of As in tailings.

Stabilization/solidification (S/S) is a technique which can eliminate or reduce the hazards of solid waste by using a binder to physically encapsulate and chemically immobilize the hazardous components in the solid waste [17,18]. It has a series of advantages, such as the ability to control the content of As quickly, low treatment cost, and simple process operation [19]. Ordinary Portland cement (OPC) is a typical binder, and the calcium silicate hydrate (C-S-H) gel generated by the OPC hydration process can effectively adsorb or ion exchange with As. Furthermore, the alkaline environment is also favorable to promote the generation of As precipitation [20]. However, the heavy use of OPC increases the treatment cost, energy consumption, and CO_2_ emissions [21]. Therefore, it is necessary to develop an efficient and environmentally friendly solution for S/S of As. This article is based on adding no alkali activator or any foreign additives, using low OPC clinker and TT for S/S of As; calcium hydroxide (Ca(OH)_2_) generated by OPC hydration can also activate TT [14]. Theoretically, it can maintain a certain strength and immobilization efficiency, and can greatly reduce the price and environment cost. In addition, 3CaO·Al_2_O_3_ (C_3_A) in OPC will generate ettringite in the presence of gypsum (CaSO_4_·2H_2_O) and Ca(OH)_2_ [22]. Ettringite is considered to be an ideal substance for S/S of As, and it mainly undergoes chemical substitution with As [23]. This study also comprehensively assesses the environmental durability to S/S of As. In natural environments, pH and temperature are important factors affecting the leaching of As in the encapsulator [24,25]. Under acidic conditions, the As precipitates in the encapsulator may be dissolved and re-released into the environment. The hydration products C-S-H gel and ettringite will also be affected [26,27]. There is a certain relationship between temperature and microstructure changes in the encapsulator, which would lead to a decrease in the performance for S/S of As [27].

In this work, we analyzed TT and investigated the compressive strength and leach property of As in the encapsulator at different OPC additions by the technology of S/S. The main species and stabilization mechanism of As in the encapsulator was also determined by various characterization tests. Durability studies were also performed for the encapsulator with different pH and temperature.

## 2. Materials and Methods

### 2.1. Materials and Experimental Procedure

The TT used in this study come from Gejiu, Yunnan Province, China, which enjoys the reputation of the word capital of tin—it has a very long mining history. There are a lot of harmful elements such as As in the TT. OPC was purchased from a factory in Zhengzhou City, Henan Province. The chemical compositions of OPC and TT were measured by X-ray fluorescence analysis (XRF), as shown in Table 1. In order to explore the effect on S/S of As, OPC and ground TT were mixed in different proportions according to the scheme in Table 2. The percentage of OPC was increased from 25 wt% to 75 wt%, and the water–binder ratio was adjusted to 0.28 according to the actual situation. Then, the mixed slurry was poured into a 40 mm × 40 mm × 40 mm plastic mold and placed at room temperature for 24 h. Then, the hardened slurry was demolded, cured to the corresponding age in a curing box at 90% humidity and 20 °C, and its compressive strength and leaching concentration of As tested. The chemical reagents nitric acid (HNO_3_), glacial acetic acid (CH_3_COOH), and sodium hydroxide (NaOH) used in this experiment were obtained from Shanghai Sinopharm Chemical Reagent Factory.

### 2.2. Leaching Tests

The leaching test was performed according to the TCLP Standard [28], and the ratio of sample to leachate was kept at 1 kg/20 L. An aqueous solution of acetic acid with pH maintained at 2.88 ± 0.05 was used as the leachate, and the pH was adjusted with HNO_3_ or NaOH. The solution was turned over at 30 rmp for 18 h at room temperature, and the pH of the solution after flipping was measured. The leachate was filtered through a 0.45 μm microporous membrane. Finally, the As leaching concentration was measured after acidifying with HNO_3_ to pH < 2. To better evaluate the environmental durability of the encapsulator, the samples were immersed in acidic solutions of pH 0–5 to investigate the effect of pH on the leachability of As, while the same samples were immersed in solutions with temperature gradients of 20–60 °C. As leaching concentrations were tested after 7 days.

### 2.3. Analysis and Characterization

**Figure 1:**
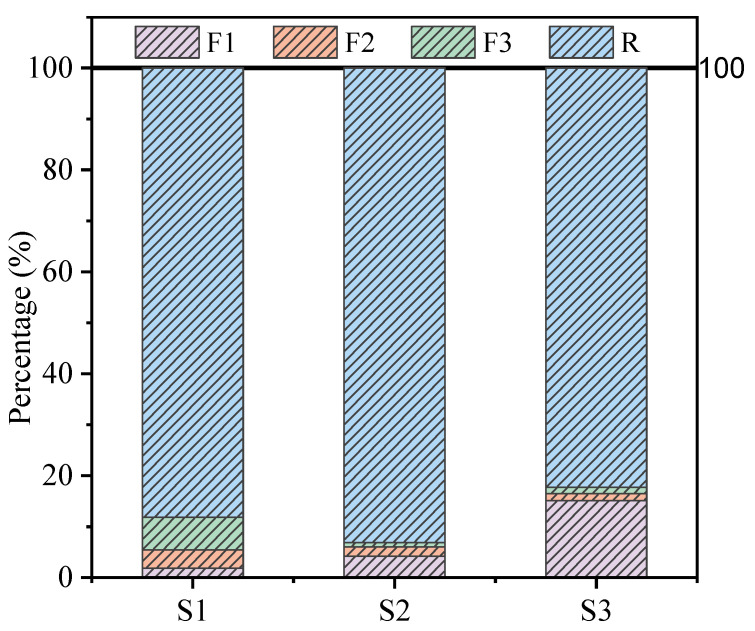
The percentage of each fraction of As in TT extracted by the BCR sequential extraction procedure.

A pH meter (Mettler FB28, benchtop acidity meter) was used to measure the weak alkalinity of the TT, pH = 8.64. The compressive strength of the samples at the corresponding age was measured by a digital pressure tester. The concentration of As in the solution after leaching the encapsulator was measured by inductively coupled plasma emission spectrometry (ICP-OES). Chemical speciation of As in TT obtained by European Community Bureau of Reference (BCR) sequential extraction analysis is presented in Figure 1, in which As is divided into acid soluble/exchangeable (F1), reducible-fraction (F2), oxidizable-fraction (F3), and residual (R) [29]. The physical phase composition of OPC, TT, and samples was determined by X-ray diffraction (XRD), setting the scan range to 5–70° and scan speed to 5°/min. Analysis of sample morphology using Scanning electron microscopy (SEM, Hitachi Regulus8100). The elemental composition of the sample was measured and analyzed by Energy dispersive spectroscopy (EDS). The functional groups of the samples were measured by Fourier transform infrared spectroscopy (FTIR) with a set scan range of 4000–400 cm^−1^, a resolution of 4 cm^−1^, and 16 scans. The samples were tested by X-ray photoelectron spectroscopy (XPS, Thermo Fisher ESCALAB XI+) to analyze the binding energy, valence, and chemical shifts of the relevant As elements.

## 3. Results and Discussion

### 3.1. X-ray Diffraction Analysis of TT and Sample

**Figure 2:**
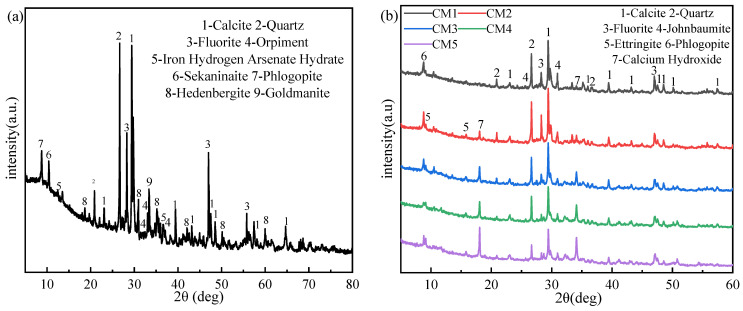
XRD spectra of tin tailing (**a**) and sample of CM1 to CM5 (**b**).

Figure 2a is the XRD spectra of TT, and the main minerals are calcite (CaCO_3_), quartz (SiO_2_), fluorite (CaF_2_), and hedenbergite (CaFe [2Si_2_O_6_]). In addition, orpiment (As_2_S_3_, PDF#24-0075) and iron hydrogen arsenate hydrate (PDF#26-0784) were also detected. Fe(H_2_AsO_4_)_3_·H_2_O is the phase formed by the oxidation of As and sulfur minerals in TT. Other phases, such as sekaninaite (Fe_2_Al_4_Si_5_O_18_) and phlogopite (KMg_3_(Si_3_Al)O_10_(OH)_2_) also correspond to the major elements in the chemical composition.

In order to understand the change process of As species after curing, the phase composition of the sample was analyzed by XRD, as shown in Figure 2b. Except for the presence of calcite (CaCO_3_, PDF#47-1743), quartz (SiO_2_, PDF#46-1045) and fluorite (CaF_2_, PDF#35-0816) phases in the TT, obviously, the mineral species of As changed before and after the S/S treatment with the appearance of johnbaumite (Ca_5_(AsO_4_)_3_(OH), PDF#33-0265), which indicates that the As species in the TT underwent chemical reactions during solidification. Fe(H_2_AsO4)_3_·H_2_O from TT released Fe^3+^ and H_2_AsO^4−^ in water, and produced Ca_5_(AsO4)_3_(OH) in the presence of Ca^2+^ and OH^−^, the transformation process of As is shown below:

(7) $$\mathrm{Fe}{\left(H_{2}{\mathrm{AsO}}_{4}\right)}_{3}\cdot H_{2}{O+\mathrm{OH}}^{-}\to {\mathrm{Fe}}^{3+}{+3H}_{2}{\mathrm{AsO}}_{4}^{-}{+H}_{2}O$$

(8) $$H_{2}{\mathrm{AsO}}_{4}^{-}{+2\mathrm{OH}}^{-}\to {\mathrm{AsO}}_{4}{}^{3-}{+H}_{2}O\;$$

(9) $${\mathrm{Fe}}^{3+}{+3\mathrm{OH}}^{-}\to \mathrm{Fe}{\left(\mathrm{OH}\right)}_{3}$$

(10) $${5\mathrm{Ca}}^{2+}{+\mathrm{OH}}^{-}{+3\mathrm{AsO}}_{4}{}^{3-}\to {\mathrm{Ca}}_{5}{\left({\mathrm{AsO}}_{4}\right)}_{3}\left(\mathrm{OH}\right)$$

**Figure 3:**
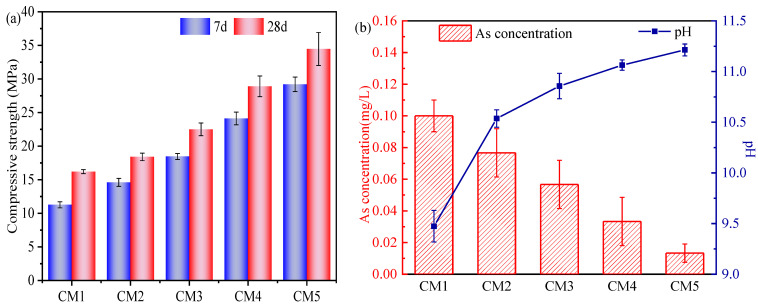
(**a**) Compressive strength of samples (CM1-CM5) after 7 and 28 days in a curing box at 90% humidity and 20 °C, (**b**) the leaching concentration of As and pH after curing 28 days.

It was shown that the diffraction peaks around 30° are related to the formation of C-S-H gels which are usually in an amorphous state, so that no distinct characteristic peaks are observed [30]. A distinct calcium hydroxide (Ca(OH)_2_, PDF#44-1481) diffraction peak can also be observed, which is due to the reaction of CaO in the cement with water to generate Ca(OH)_2_ [31]. With the increase of OPC content, the amount of Ca(OH)_2_ generated increases, and the intensity of the diffraction peak increases, which is also consistent with the increase of pH value of the leaching solution in Figure 3b. The appearance of ettringite is due to the chemical reaction between C_3_A with a small amount of gypsum (CaSO_4_·2H_2_O) doped in the mixture, which is beneficial to the S/S of As. The peak intensity of ettringite increases with increasing OPC content, indicating that more ettringite was generated, which also explains the increasing trend of compressive strength. Ca(OH)_2_ can also be used as an alkali activator to activate the TT [32], and with the increase of OPC content, ettringite has a tendency to gradually increase.

### 3.2. Compressive Strength and Leaching Test

As shown in Figure 3a, the 7-day and 28-day compressive strengths of sample showed a sequential increase with the increase of cement content. At the highest content of TT, the compressive strengths of sample for 7 days and 28 days respectively were 11.3 MPa and 16.2 MPa, and the presence of less active substances in TT was not conducive to the development of compressive strength. The compressive strengths of sample for 7 days and 28 days were 28.9 MPa and 34.7 MPa when the TT content was the lowest. The increase in cement content promoted the formation of more C-S-H gels. These C-S-H gel phases were filled in the voids between the hydration products, which increases the structure compactness and greatly improves the compressive strength [33,34]. The formation of ettringite is beneficial to improve the early compressive strength [35]. In addition, ettringite also has a certain degree of swelling, which can reduce the porosity of the encapsulator and further improve the compactness of the structure [36].

The trend of As leaching concentration obtained from the experiment is shown in Figure 3b, where the concentration of As in the solution is inversely proportional to the content of TT. With the decrease of TT, the leaching concentration of As decreased from 0.1 mg/L to 0.01 mg/L, and the pH value increased from 9.47 to 11.21. The presence of many alkaline substances in the cement, which generated OH^−^ during the hydration process, explains the increase of the pH value of the leachate after 28 days, and facilitated the formation of Ca-As compound precipitation [37], thus proving the reason for the decrease of As leaching concentration. In addition, OPC hydration products such as C-S-H gels have adsorption effects on As, and the hydroxide colloids formed by Fe^2+^ and Fe^3+^ in alkaline environment can adsorb different forms of As-containing compounds on the surface or encapsulate them in the colloids [38].

### 3.3. Fourier Transform Infrared Spectroscopy Analysis

**Figure 4:**
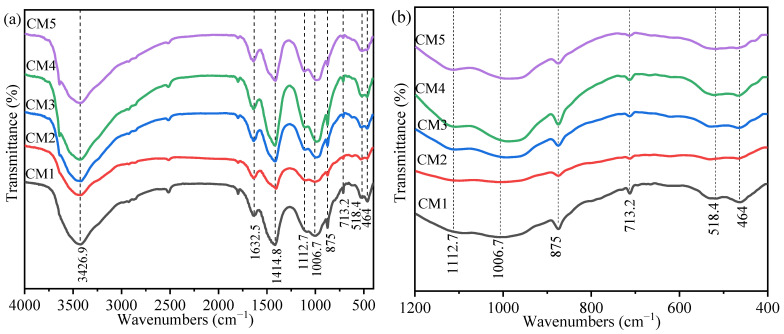
FTIR spectra of sample CM1 to CM5 (**a**,**b**).

Figure 4 shows the FTIR spectra of CM1–CM5 with similar trends for all the curves. The functional groups corresponding to the FTIR absorption peaks are summarized in Table 3. The peak at 3426.9 cm^−^^1^ is due to the -OH stretching vibration from water in the free state of the sample. The peak at 1632.5 cm^−1^ is H-O-H bending vibration, indicating the presence of C-S-H. The peak at 1414.8 cm^−1^ is the stretching vibration caused by the O-C-O of the carbonate. The peaks at 1112.7 cm^−1^ and 713.2 cm^−1^ are the v3 and v4 vibrations of SO_4_^2−^, which implies the presence of ettringite. The peaks at 1006.7 cm^−1^ and 518.4 cm^−1^ are the v3 and v4 vibrations of SiO_4_^2−^. The peak at 875 cm^−1^ is attributed to the As-O stretching vibration, which corresponds to the formation of arsenic–oxygen compounds in Figure 2b. It is further verified that As in TT exists in the form of precipitation [39,40]. The peak at 464 cm^−1^ indicates the Si-O bending vibration.

### 3.4. X-ray Photoelectron Spectroscopy Analysis

**Figure 5:**
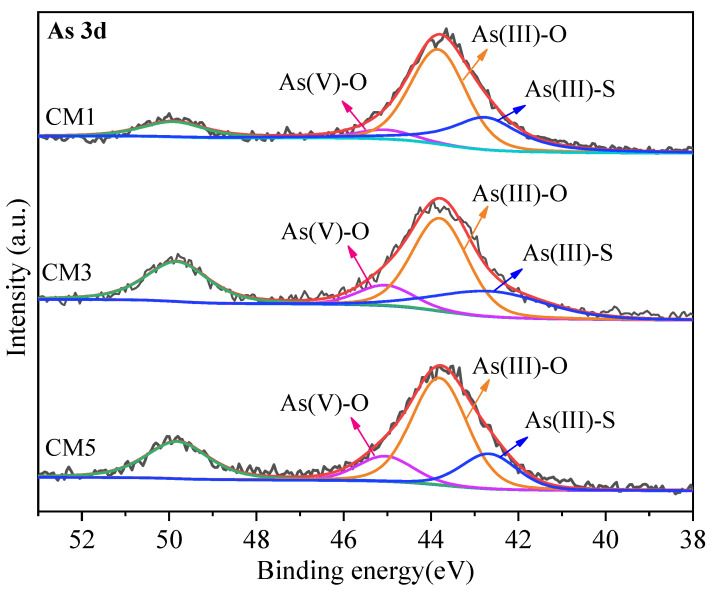
XPS spectra of CM1, CM3, and CM5 (**As 3d**).

From the XPS spectrum of Figure 5, it was observed that As(III)-O dominates in the sample, exists in an amorphous state, and forms a precipitate with Ca^2+^, the transformation process of As is shown below:

(11) $${2H}_{3}{\mathrm{AsO}}_{3}{+3\mathrm{OH}}^{-}\to {\mathrm{AsO}}_{3}^{3-}{+3H}_{2}O$$

(12) $${3\mathrm{Ca}}^{2+}{+2\mathrm{AsO}}_{3}{}^{3-}\to {\mathrm{Ca}}_{3}{\left({\mathrm{AsO}}_{3}\right)}_{2}$$

As in TT is mainly present in the form of As_2_S_3_ and FeAsS species in Figure 2. The presence of As(V)-O, As(III)-O, and As(III)-S can also be observed in the XPS spectra of the treated TT in Figure 5. During the process from CM1 to CM5, As(III)-S decreased from 32.5% to 21.0% and As(V)-O increased from 6.1% to 15.1%, which indicates that As_2_S_3_ underwent dissolution and finally converted to As(V), and the oxidation process was very slow [9]. The percentage of As(III)-O was also changing, indicating that oxidation of As_2_S_3_ first formed As(III)-O and then further converted to As(V)-O. Although the solubility of As_2_S_3_ in water is small, the equation for the hydrolysis is as follows:

(13) $${\mathrm{As}}_{2}S_{3}{+4H}_{2}O\to {2\mathrm{HAsO}}_{2}{+3H}_{2}S$$

Both HAsO_2_ and H_2_S react with alkali, and the reaction equations are as follows:

(14) $${\mathrm{HAsO}}_{2}{+\mathrm{OH}}^{-}\to {\mathrm{AsO}}_{2}{}^{-}{+H}_{2}O\;$$

(15) $$H_{2}{S+2\mathrm{OH}}^{-}\to S^{2-}{+2H}_{2}O\;$$

In the presence of O_2_, the oxidation Equation (14) of arsenite is as follows:

(16) $${2\mathrm{AsO}}_{2}{}^{-}{+O}_{2}{+4\mathrm{OH}}^{-}\to {2\mathrm{AsO}}_{4}{}^{3-}{+2H}_{2}O$$

The XPS spectra show that the As species are dominated by As(III)-O. In addition to As_2_S_3_, other As sulfides and As_2_O_3_ in the tailings can be converted to arsenite during the synthesis process [49].

### 3.5. SEM-EDS Analysis

**Figure 6:**
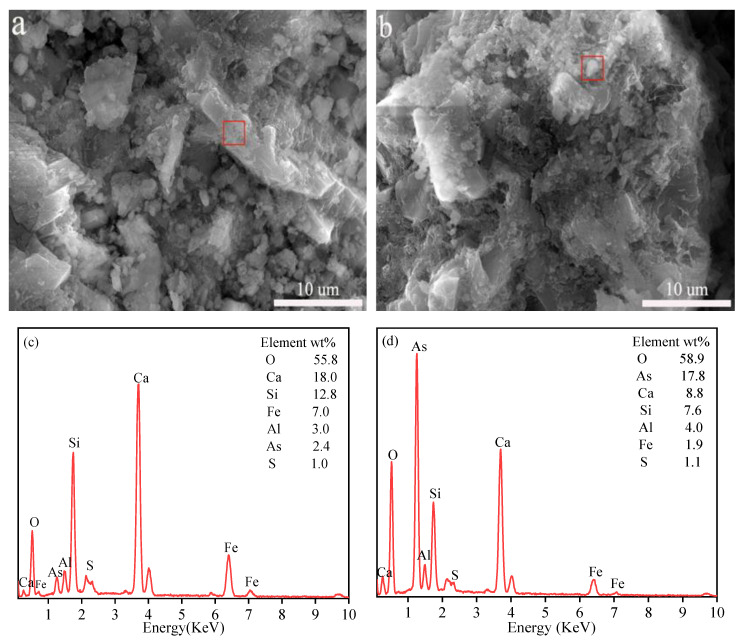
SEM images: (**a**) CM1 (**b**) CM5; and EDS analyses of elements composition: (**c**) CM1 (**d**) CM5.

The presence of many white irregular amorphous C-S-H gels in Figure 6b is considered to be the main product of cement hydration; it is capable of S/S of As by physical adsorption, interlayer symbiosis, and ion exchange [50]. In addition, some needle-like crystal organization is typical for the shape of ettringite; it has a strong heavy metal immobilization ability, and anionic groups such as arsenate ions are able to ion exchange with SO_4_^2−^ in ettringite for stabilization [31]. EDS in Figure 6c results show the presence of O, As, Ca, Si, Al, and Fe elements, indicating that As combines with Ca and Fe to produce the corresponding precipitates. Element S was found in Figure 6d, indicating that a portion of arsenic sulfide was still present and was not involved in the reaction, which is consistent with the results in Figure 5.

### 3.6. Environmental Durability Assessment

The environmental durability assessment is to study the influence of external factors on the performance of S/S of As. This experiment mainly simulated the influence of two important factors, pH and temperature of acidic conditions, and to study their effects on the leaching behavior of As.

#### 3.6.1. Effect of pH on the Leaching Behavior of As

**Figure 7:**
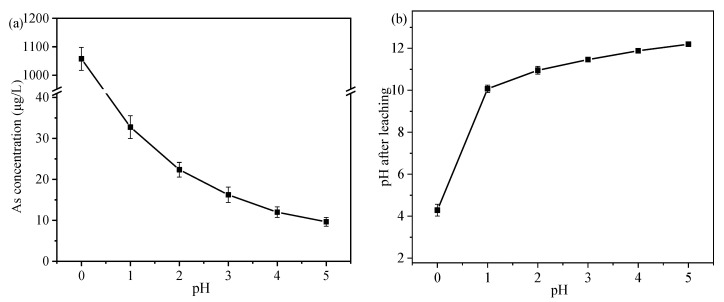
(**a**) The influence of different pH on the leaching concentration of As, (**b**) the change of pH after leaching.

The cured samples were dipped into the acid solution at a ratio of 1:10 (g/mL), the different solutions of pH 0–5 adjusted with HNO_3_, and the leaching concentration of As was tested after 7 days. The results are shown in Figure 7a, the leaching concentration of As gradually decreased with the increase of pH, the concentration of As decreased from 1056.67 μg/L to 32.76 μg/L when the pH was between 0 and 1, and then the concentration of As showed a slow downward trend and finally dropped to 9.65 μg/L. The study showed that the release of As from the tailings was favorable under acidic environment. Especially in the strong acidic environment with pH = 0, the stability of As-S, Fe-As, and Ca-As species in the TT will substantially reduce [26,51]. Moreover, the structure of C-S-H and ettringite will be destroyed, leading to an increase of As leaching.

Due to the presence of many alkaline substances in the encapsulator, mainly Ca(OH)_2_ released OH^−^ to the solution, leading to an increase of pH in Figure 7b. In a solution with an initial pH of 0, the OH^−^ released by the encapsulator cannot completely consume the H^+^ in the solution, resulting in the solution remaining acidic (pH = 4.29). It was shown that the stability of Fe-As species was favored under weakly acidic conditions [52], but Ca-As species dominated the whole encapsulator and Ca-As species were poorly stable under acidic conditions [53]. As a result, the concentration of As in the solution decreased dramatically. The change of As concentration at different pH can be divided into two stages: the first stage in the range of pH from 0 to 1, the As concentration decreases sharply and the final solution is weakly acidic, and the second stage in the range of pH from 1 to 5, the As concentration decreased slowly and the change gradually stabilized and the final solution is alkaline (pH = 10.07~12.19). Therefore, this sequester still has good results in S/S of As at pH > 1.

#### 3.6.2. Effect of Temperature on the Leaching Behavior of As

**Figure 8:**
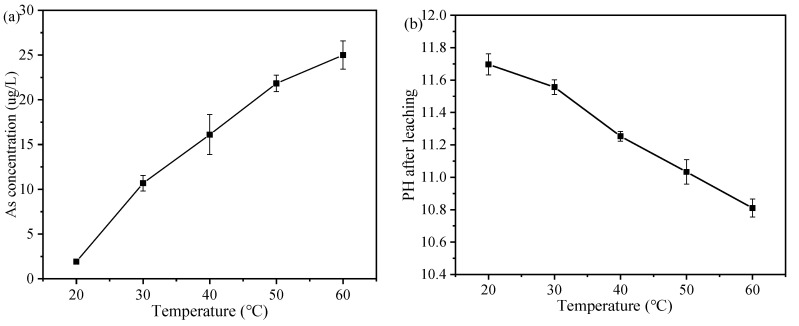
(**a**) The influence of different temperature on the leaching concentration of As, (**b**) the change of pH after leaching.

The pH value was adjusted to 3 with HNO_3_, and the ratio of sample to leachate and leaching time were kept constant. The results are shown in Figure 8a, with the increase of temperature, the leaching concentration of As increased from 1.91 μg/L to 24.99 μg/L, and the trend of change was stable. The influence of leaching temperature on As leaching concentration is mainly reflected in the change of diffusion coefficient [25], the change of pores and structure of the encapsulator, and the formation and development of hydration products [54]. Leaching is the process by which substances in the solid phase are released into the liquid phase through the surface. The higher the temperature, the faster the diffusion rate of As, which leads to an increase in the concentration of As in the liquid phase. We also found that the amount of change in As concentration per unit temperature decreases as the temperature increases. It was shown that C-S-H and ettringite dissolve at high temperatures [55]. The change in temperature is also reflected in the effect on the pH of the solution; Figure 8b shows the pH value gradually decrease with increasing temperature, which is mainly due to the decrease in the solubility of the hydration product Ca(OH)_2_ with increasing temperature [56]. The decrease in alkalinity of the solution also affects the stability of the Ca-As species, leading to the release of As from the precipitation.

#### 3.6.3. SEM Analysis

**Figure 9:**
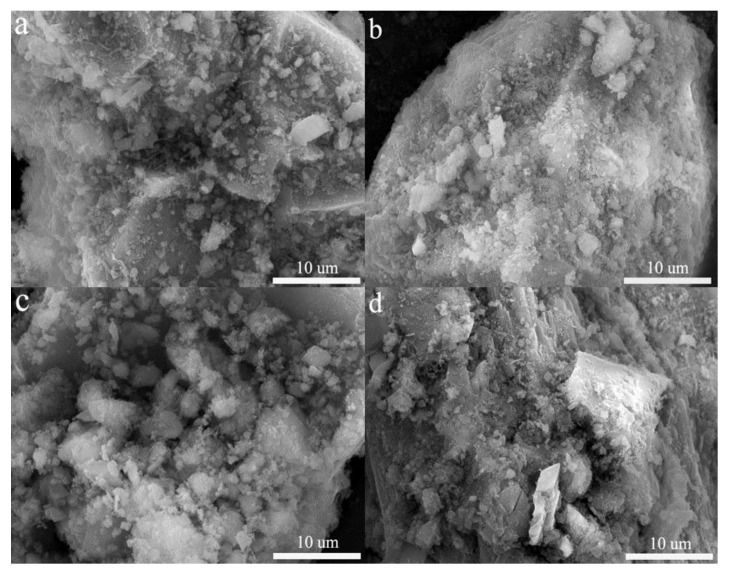
SEM images different pH and temperature: (**a**) pH = 1; (**b**) pH = 5; (**c**) temperature = 20 °C; (**d**) temperature = 60 °C.

Figure 9 shows the effect of different pH and temperature on the surface morphology of the encapsulator. These amorphous white substances are mainly C-S-H gels and cementitious materials, and compared to pH = 5 (Figure 9b), at pH = 1 (Figure 9a) it can be observed that the surface is smoother and less dense, and the C-S-H gels and cementitious materials gathered on the surface undergo dissolution, leading to the re-release of the internal As [57]. The dissolution of these materials releases OH^−^ and raises the pH of the solution, which is consistent with the results in Figure 7. The effect of temperature on the encapsulator is also mainly reflected in the change of C-S-H gel and cementitious materials, compared with 20 °C (Figure 9c), a decrease of C-S-H and cementitious materials aggregated on the surface is clearly observed at 60 °C (Figure 9d). The temperature has a catalytic effect on the chemical reaction, and accelerates the erosive effect of H^+^ on C-S-H and cementitious materials [58]. Furthermore, we can observe the appearance of cracks and an increase of surface porosity, where excess heat at higher temperatures can cause thermal damage to the encapsulant. We can observe that cracks appear and the surface pore increase, the excess heat is generated at higher temperatures, thermal damage to the encapsulator can occur.

### 3.7. Mechanism of S/S Arsenic

**Figure 10:**
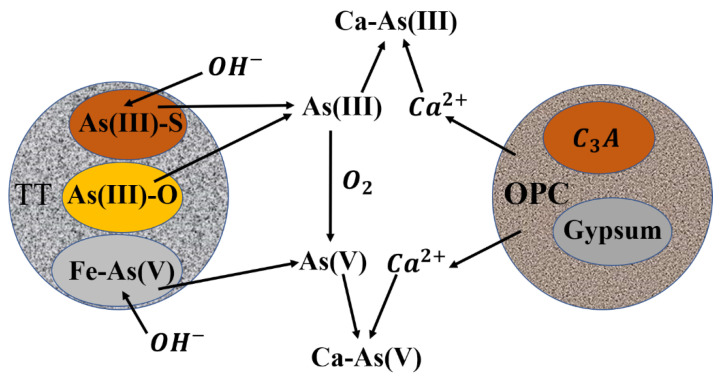
The mechanism of S/S of As in the encapsulator.

TT is a hazardous solid waste containing As, and if not treated, the internal As will be released into the surrounding environment under the action of natural weathering. The As species in TT are complex, mainly As(III)-S, Fe-As(V), and amorphous As(III)-O species. The As in TT undergoes oxidation reaction, and the As-containing phases As_2_S_3_ and Fe(H_2_AsO_4_)_3_·H_2_O can be observed in the Figure 2. The reaction conversion mechanism of As is shown in Figure 10. The hydration process of OPC releases OH^−^, which results in the whole system being alkaline, and the AsO_4_^3−^ released by the Fe(H_2_AsO_4_)_3_·H_2_O reaction combines with Ca^2+^ and OH^−^ to form Ca_5_(AsO_4_)_3_(OH) and Ca_3_(AsO_4_)_2_. A small fraction of Fe^3+^ present in the system can generate FeAsO_4_ precipitate with AsO_4_^3−^, and also generate Fe(OH)_3_ colloid with OH^−^, but in this system, mainly Ca-As species are still dominant. The solubility of As_2_S_3_ in water is small, but the hydrolysis products HAsO_2_ and H_2_S can easily react with OH^−^, therefore promoting the hydrolysis of As_2_S_3_. The As_2_S_3_ and amorphous As(V)-O species provide As(III) and Ca^2+^ to form Ca-As(III) precipitates. Furthermore, the hydration products C-S-H and ettringite also have excellent ability for S/S of As, C-S-H by physical adsorption, ion exchange, and interlayer symbiosis, and ettringite mainly through ion exchange of arsenate group and SO_4_^2−^ in the structure. FTIR curves of Si-O, AS-O, and H-O-H indicate that C-S-H and ettringite have ion exchange with As underwent ion exchange in 0. Amorphous C-S-H and needle-tipped ettringite can be clearly observed in 0 and elemental As was also detected by EDS.

The assessment of S/S of As ability should consider the influence of external conditions on the encapsulator. Lowering pH and increasing temperature contribute to the release of As, mainly by promoting the dissolution of As immobilized phase and As immobilized materials, as shown in Figure 9, and pH has a greater effect on As immobilization than temperature. The effects of pH and temperature on the long-term performance of the encapsulator and the effects of other factors on S/S of As need further research. Therefore, it is necessary to employ a systematic and comprehensive assessment of the long-term performance of the encapsulator under various factors in the future to ensure the safety and long-term application of the S/S technology.

## 4. Conclusions

In this study, S/S treatment of arsenic in TT was performed using OPC and the leaching concentration of arsenic after 28 days was 0.01 mg/L, and it was reduced to a safe range. The leaching concentration of arsenic was reduced to a safe range. During the S/S treatment, OH^−^ produced by OPC hydration promoted the dissolution of As(III)-S species and Fe-As(V) species and combined with Ca^2+^ to form Ca-As(V) precipitates, and amorphous As(III)-O-containing species also formed Ca-As(III) precipitates with Ca^2+^, and the dissolved As(III) underwent oxidation reactions, leading to an increase in As(V). The C-S-H gel generated by OPC hydration and ettringite with arsenate group also underwent adsorption and ion exchange to achieve S/S of As effect. In addition, the encapsulator showed good durability in a wide range of acidic environments and temperatures.

## Figures and Tables

**Table 1:** Chemical compositions of OPC and TT (wt%).

Material	Al_2_O_3_	CaO	Fe_2_O_3_	K_2_O	MgO	P_2_O_5_	SiO_2_	SO_3_	LOI
OPC	7.1	41.3	6.2	0.3	2.8	0.09	24.5	0.05	17.66
TT	6.2	28.5	16.4	1.1	0.08	0.06	31.7	2.4	13.56

LOI: Loss on ignition.

**Table 2:** Mixture formulations (wt%) for OPC and TT.

Sample	Mixture Ratio (wt%)		Water Cement Ratio (W/C)
	TT	OPC	
CM1	75	25	0.28
CM2	66.7	33.3	
CM3	50	50	
CM4	33.3	66.7	
CM5	25	75	

**Table 3:** Peaks detected by FTIR spectroscopy and the corresponding functional groups.

Wavenumbers (cm^−1^)	Functional Group	Types of Vibration	Reference
3426.9 cm^−1^	Free H_2_O	stretching vibration	[30]
1632.5 cm^−1^	H_2_O in C-S-H	bending vibration	[41]
1414.8 cm^−1^	O-C-O	stretching vibration	[42]
1112.7 cm^−1^	SO_4_^2−^	v3 vibrations	[43]
1006.7 cm^−1^	SiO_4_^2−^	v3 vibrations	[44]
875.0 cm^−1^	As-O	stretching vibration	[45,46]
713.2 cm^−1^	SO_4_^2−^	v4 vibrations	[16]
518.4 cm^−1^	SiO_4_^2−^	v4 vibrations	[47]
464.0 cm^−1^	Si-O	bending vibration	[48]

## Data Availability

Not applicable.
